# The COVID-19 pandemic and disruptions in a district quality improvement initiative: Experiences from the CLEVER Maternity Care programme

**DOI:** 10.4102/safp.v64i1.5359

**Published:** 2022-03-30

**Authors:** Sarie J. Oosthuizen, Anne-Marie Bergh, Antonella Silver, Refilwe E. Malatji, Vivian Mfolo, Tanita Botha

**Affiliations:** 1 Department of Family Medicine, Faculty of Health Sciences, University of Pretoria, Pretoria, South Africa; 2 Research Centre for Maternal, Fetal, Newborns and Child Health Care Strategies, Faculty of Health Sciences, University of Pretoria, Pretoria, South Africa; 3 SAMRC Research Unit for Maternal and Infant Health Care Strategies, Faculty of Health Sciences, University of Pretoria, Pretoria, South Africa; 4 District Clinical Specialist Team, Tshwane District Health Services, Tshwane, South Africa; 5 Department of Statistics, Faculty of Natural and Agricultural Sciences, University of Pretoria, Pretoria, South Africa

**Keywords:** COVID-19, health-systems readiness, maternity services, quality, CLEVER Maternity Care, working environment, communication

## Abstract

**Background:**

Many health systems were poorly prepared for the coronavirus disease 2019 (COVID-19) pandemic and found it difficult to protect maternity and reproductive health services. The aim of the study was to explore the influence of the COVID-19 pandemic on the ability of maternity healthcare providers to maintain the positive practices introduced by the CLEVER Maternity Care programme and to elicit information on their support needs.

**Methods:**

This multimethod study was conducted in midwife-led obstetric units (MOUs) and district hospitals in Tshwane District, South Africa and included a survey questionnaire and qualitative reports and reflections by the CLEVER implementation team. Two five-point Likert-scale items were supplemented by open-ended questions to provide suggestions on improving health systems and supporting healthcare workers.

**Results:**

Most of the 114 respondents were advanced midwives or registered nurses (86%). Participants from MOUs rated the maintenance of quality care practices significantly higher than those from district hospitals (*p* = 0.0130). There was a significant difference in perceptions of support from the district management between designations (*p* = 0.0037), with managers having the most positive perception compared with advanced midwives (*p* = 0.0018) and registered nurses (*p* = 0.0115). The interpretation framework had three main themes: working environment and health-system readiness; quality of patient care and service provision; and healthcare workers’ response to the pandemic. Health-facility readiness is described as proactive, reactive or lagging.

**Conclusion:**

Lessons learned from this pandemic should be used to build responsive health systems that will enable primary healthcare workers to maintain quality patient care, services and communication.

## Introduction

The novel coronavirus disease 2019 (COVID-19) spread rapidly throughout the world at the beginning of 2020. On 30 January 2020, the World Health Organization (WHO) declared the outbreak a Public Health Emergency of International Concern (PHEIC) under the International Health Regulations (2005)^1^ and South Africa recorded its first case on 05 March 2020.^2^ Countries had to move quickly to draft the necessary legislation, review policies and find the finances required to implement the WHO recommendations.

Health systems in low- and middle-income countries (LMICs) were poorly prepared for the pandemic and found it difficult to shift from policy to implementation, which limited their ability to achieve the necessary emergency responses.^3,4^ They found it a struggle to adhere to the requirements emanating from the pandemic and to protect essential activities, including district maternity and reproductive healthcare services.^4,5,6,7^ Weak infrastructure and overload contributed to suboptimal patient safety and infection control measures.^7,8^ Senior managers and policymakers did not communicate early and clearly enough,^3^ leading to ineffective planning and preparation in facilities where many healthcare professionals were searching for information themselves.^9^ This lack of collaboration in decision making impacted facilities’ ability to provide care and left healthcare workers (HCWs) feeling unsafe and undervalued.^3,9^ Healthcare workers also reported feeling overwhelmed and experiencing acute stress, anxiety and depression.^10^ The lack of coordination and slow implementation at facilities caused lapses in occupational health and safety on the part of health workers.^11^

Tshwane Health District is mainly an urban district in Gauteng and 73% of the population utilise public healthcare services. When the first COVID-19 cases were reported in Tshwane we were in the process of rolling out and monitoring the effects of the CLEVER Maternity Care outreach programme in all five district hospitals (DHs) and all 10 midwife-led obstetric units (MOUs) in the district. The CLEVER Maternity Care is a package designed to strengthen the health system and to facilitate the improvement of the quality of obstetric care with regard to skills and respectful care. The package included weekly and monthly outreach visits with mentoring and constructive feedback, especially during ward rounds. It was first piloted in five MOUs and demonstrated a significant decrease in fresh stillbirths, birth asphyxia and meconium aspiration.^12^ The further rollout to the rest of the public primary healthcare facilities in the district was embedded in the work of the district clinical specialist team (DCST) with additional support from an implementation team.

The hard lockdown at the end of March 2020 brought programme activities to a standstill and the weekly intensive engagement visits over a period of 3 months – an integral part of the CLEVER programme – could not be completed for the last five MOUs. In July 2020 support visits to the CLEVER facilities were resumed in a different manner, with more visits scheduled for the facilities that had more needs and issues as a result of the pandemic. One busy district hospital had been converted to a COVID-19 designated hospital and pregnant women and women in labour were diverted to other facilities. Another district hospital, for example, saw an increase of 33% in deliveries from April to December 2020, with no additional staff provided (unpublished information). To complement the activities of the CLEVER implementation team, Tshwane DCST members visited facilities to assess their readiness for the pandemic and identify health-system gaps where strengthening was needed.

The observations of the two teams regarding the provision of healthcare services in Tshwane were similar to reports published globally and in South Africa.^13,14^ When the COVID-19 designated district hospital reached full capacity during the peak of the first wave of the pandemic, the overflow of COVID-confirmed patients and persons under investigation (PUIs) reached the rest of the healthcare facilities. Although all facilities had received directives to prepare an isolation space and other equipment, they were at different levels of readiness to receive these patients. Many complaints were received about shortages of personal protective equipment (PPE), masks and hand sanitisers and midwives being reluctant or refusing to work without the necessary resources.

Very soon it became clear that the pandemic was disrupting the functioning of health systems that could potentially affect HCWs’ resilience to continue providing quality care. As the provision of maternity services was also impacted, we identified the need for more structured feedback from maternity care providers on their perceptions and experiences of changes in the quality-of-service provision, including the practices introduced through the CLEVER Maternity Care programme. Their account of changes in patient care and the way these had affected their ability to continue with the positive practices introduced by CLEVER Maternity Care could highlight the support needs to be addressed by the CLEVER team, the DCST and the district management team.

## Study design and research methods

**FIGURE 1:**
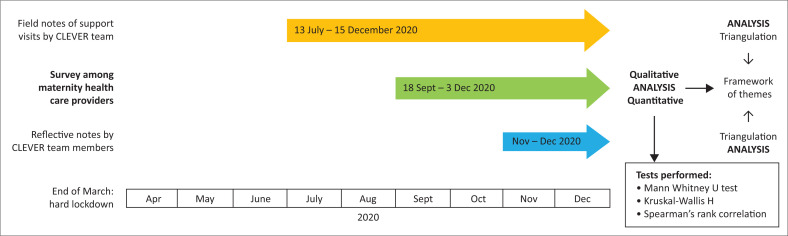
Multimethod research design and timeline of data collection and analysis.

This study had a multimethod design that included the following: (1) a survey questionnaire with closed- and open-ended questions; (2) the CLEVER implementation team’s field notes of HCWs’ reports of changes in service provision during facility visits after the initial lockdown had prevented further rollout of and support for the programme; and (3) reflective notes by the CLEVER team on the pandemic readiness of the different health facilities. The CLEVER research and implementation team comprised a family physician, an obstetrician, an advanced midwife, a primary healthcare nurse and a social scientist. Figure 1 is a graphic representation of the research design and the timeline of data collection and analysis.

The survey was the main focus of the study and was conducted from 18 September to 03 December 2020 amongst healthcare providers working in maternity in 14 of the 15 CLEVER facilities. The converted COVID-19 designated district hospital was excluded. The additional pressure brought about by the pandemic made it impossible to sample the total population or a randomised group of eligible participants. Therefore, all HCWs on duty at the time of a visit by CLEVER team members were asked to complete the survey voluntarily and anonymously. Only HCWs employed at the particular healthcare facility for at least one year were included. This criterion excluded student nurses, community service nurses placed at a facility for a single year and other workers who did not have sufficient experience at the facility to be able to make a meaningful comparison of changes in service provision or practice as a result of the pandemic.

### Survey tool and data collection

The urgency of getting the feedback necessitated the design of a rapid tool that would not infringe on the service responsibilities and time of HCWs. A short anonymous self-reporting questionnaire was developed and refined by the authors. It was presented to 13 HCWs who were familiar with the context of maternity care in Tshwane District to comment on the face validity of the tool with regard to format, length, content and feasibility of generating useful findings.

The questionnaire elicited demographic information on age, gender, designation and years of employment at a CLEVER facility. Two five-point Likert-scale items pertained to health-systems issues: how COVID-19 was perceived to have affected care practices that had been introduced or strengthened during the rollout of the CLEVER package (‘All care practices maintained’ to ‘Not maintained’) and how HCWs experienced the support they had received from district management for providing continued quality of care (‘Excellent’ to ‘None’). Each item was complemented by an open-ended question in which participants could, respectively, describe what practices had been maintained or not maintained or make suggestions for improvement of support. The questionnaire also contained two items related to HCWs’ perceptions of their own well-being. These results are reported separately.^15^

CLEVER team members distributed the questionnaire to HCWs during their support visits. Placing the questionnaire in a special collection envelope was considered consent to use the information.

The use of data collected from additional sources was a form of method triangulation^16^ to enhance the trustworthiness of the survey’s findings. After the hard lockdown, facility visits could only resume on 13 July 2020 and ended on 15 December 2020. After each facility visit the CLEVER team tabulated their observations and the feedback received from HCWs on their experience of changes and challenges in service provision under the following headings: date of visit; facility name; team members on the visit; training, activities and discussion; observations and notes (including reported service provision challenges); tools and needs (issues for follow-up); and number of HCWs reached. Field notes were available for 96 visits and were discussed weekly by the research team. In November and December 2020, each team member wrote reflective notes structured around the WHO health-systems building blocks^17^ on their perceptions of the pandemic readiness of each CLEVER health facility. In order to develop a better understanding of the context of each facility and to identify facilities with more challenges, they ranked facilities according to their impressions of each one’s pandemic readiness.

### Data management and analysis

Data were captured on Excel and analysed with the R statistical software package version 4.0.3.^18^ Descriptive statistics were generated (frequencies, proportions, means and medians). To measure participants’ perceptions on changes in practice and fulfilment of support needs, a score of 0–4 was allocated to the five options as follows:

- Maintenance of good practice score: All care practices maintained = 4, most = 3, some = 2, few = 1, none = 0
- Support needs score: Excellent support = 4, good support = 3, some support = 2, little support = 1, no support = 0

The median and mean scores for the two items were then calculated out of 4.

The Mann–Whitney U test was used to compare two independent groups such as demographic variables and experiences of practice change and support. When multiple groups were being compared, the Kruskal–Wallis H test was used, followed by post hoc analysis, including the Bonferroni adjustments, for cases where a significant difference was observed. In some instances, significance could not be determined because numbers in certain categories were too small. Numerical age and years of employment were used in comparison calculations and Spearman’s rank correlation was performed to establish these variables’ relationship with the maintenance of good practice and support needs scores. All significance tests were performed at a 5% level of significance.

Four members of the research team worked together on the data analysis and interpretation of the qualitative data to ensure the quality of the analysis. The framework method of analysis as described by Gale et al.^19^ was adapted to do inductive content analysis of responses to the two open-ended questions in the questionnaire. One researcher (A.-M.B.) captured all open-ended text in Excel and familiarised herself with the transcribed responses. An initial code list was generated from the data from the first 32 captured questionnaires and the responses were charted on a provisional framework document with themes, subthemes and categories. The rest of the research team considered the suitability and feasibility of the proposed analytical framework and provided further inputs into the framework structure. The analytical framework was then applied to the open-ended responses using NVivo 9.0 software. Subthemes and subcategories were further refined during the analysis. The final framework had three main themes and 10 subthemes, each with between one and nine categories.

After the analysis of the questionnaire responses, the content of the field notes and reflective notes of the CLEVER team members were analysed and compared with the themes, subthemes and categories that had emerged from the HCWs’ direct responses. The same themes and subthemes were found in these additional data sources.

The research team also developed a typology of facilities’ pandemic readiness using the WHO health-systems building blocks – leadership and governance, healthcare workforce, service delivery, financing, essential supplies and information systems^17^ – to develop criteria for dividing the CLEVER facilities into categories of pandemic readiness. The criteria were applied to each facility, based on feedback from the maternity HCWs and researchers’ field notes and reflections. The purpose of this exercise was to flesh out in more detail what kind of support each healthcare facility needed for providing essential maternity services of high quality and for maintaining the gains made through the CLEVER programme. Four members of the research team ranked all study facilities according to the identified categories. The researchers’ ranking lists were pooled to get to a readiness score out of 12 for each facility.

### Ethical considerations

The study’s proposal was approved by the Research Ethics Committee of the Faculty of Health Sciences, University of Pretoria, as an amendment to the CLEVER Maternity Care protocol 787/2018 and all methods were carried out in accordance with relevant guidelines and regulations. The Tshwane district management team supported the study and issued a letter of permission for healthcare workers to participate. Completion of the anonymous survey questionnaire was voluntary. The introductory letter in the questionnaire stated that informed consent would entail handing back the completed questionnaire to the research team.

## Results

### Study participants

A total of 135 questionnaires were received, of which 21 were excluded because of an employment period of less than one year at the facility. The responses to 114 questionnaires were eventually analysed – 65 participants from MOUs and 49 from DHs. The number of participants per MOU ranged from 4 to 11 and those per DH from 7 to 16.

**FIGURE 2:**
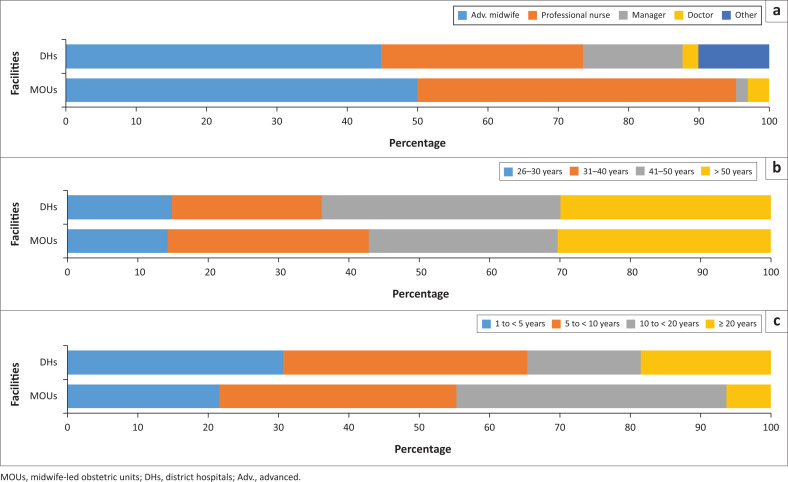
Distribution of participants according to facility type, designation, age and length of employment. (a) Distribution of participants by designation; (b) distribution of participants by age group; (c) distribution of participants by length of employment.

Most of the participants were advanced midwives or registered nurses (*n* = 97; 85.8%). Figure 2 gives a graphic depiction of the distribution of participants between MOUs and DHs by designation, age and length of period of employment. As there was only one male respondent, no analysis was performed with regard to gender. The only marked difference in terms of designation was the smaller percentage of registered nurses from DHs (28.6%) than from MOUs (44.6%). The mean age of participants was 42.63 (±10.71) years, with ages ranging from 25 to 68 years. There was no significant difference in the age of respondents in MOUs and DHs (*p* = 0.9398). Figure 2 shows the breakdown of the fairly equal distribution of participants into four age groups. The smaller number of participants in the 25–30 years group is explained by the shorter span of 6 years instead of ≥ 10 years in the other groups. The mean length of employment in a study facility was 9.42 (± 7.03) years, with the longest employment period being 26 years in one MOU and 36 in one DH. There was no significant difference in the length of employment between staff members in MOUs compared with those in DHs (*p* = 0.1778). For convenience, participants were divided into four groups on the basis of length of employment: 1 to < 5 years, 5 to < 10 years, 10 to < 20 years and ≥ 20 years. There were more participants from DHs in the 1 to < 5 years group (30.6%) than from MOUs (21.5%), as well as in the ≥ 20 years group (18.4% vs 6.2%). Midwife-led obstetric units, on the other hand had more participants in the 10 to < 20 years group (38.5% vs 16.3%).

### Perceptions on the maintenance of care practices

**FIGURE 3:**
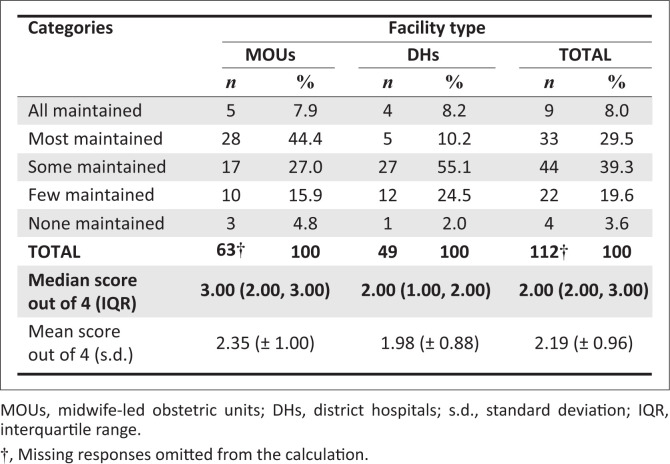
Comparison of midwife-led obstetric units and district hospitals participants’ responses regarding the maintenance of good practice.

Participants’ responses to the question whether a facility had been able to maintain all the positive changes introduced by the CLEVER Maternity Care programme are summarised in Figure 3. The median maintenance of good practice score out of 4 was significantly higher for MOU participants (3.00 [2.00, 3.00]) than for DH participants (2.00 [1.00, 2.00]) (*p* = 0.0130). The majority of responses ticked by MOU participants were ‘Most maintained’ (44.4%) and ‘Some maintained’ (15.9%), whereas for DHs the emphasis was on ‘Some maintained’ (55.1%) and ‘Few maintained’ (24.5%). Overall, the majority of participants felt that at least some or most of the practices were being maintained.

Participants’ age, designation and length of employment at their current facility did not have a significant influence on their perceptions of the maintenance of or changes in clinical practice after the advent of COVID-19 (*p* = 0.2383; *p* = 0.1974; *p* = 0.3484, respectively).

### Perceptions on the fulfilment of support needs

**FIGURE 4:**
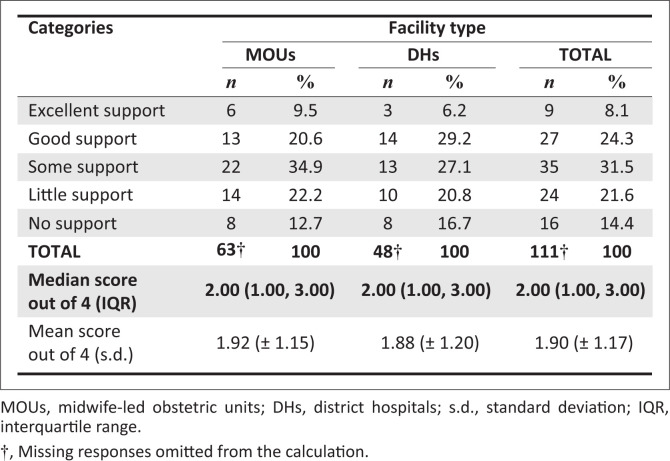
Comparison of midwife-led obstetric units and district hospitals participants’ perceptions of support received.

There was no significant difference in perceptions of support received from district management after the advent of COVID-19 between MOU and DH participants (*p* = 0.9340). A more detailed comparison is contained in Figure 4. When all 14 facilities were compared with each other there was a tendency towards a difference (*p* = 0.0764).

**FIGURE 5:**
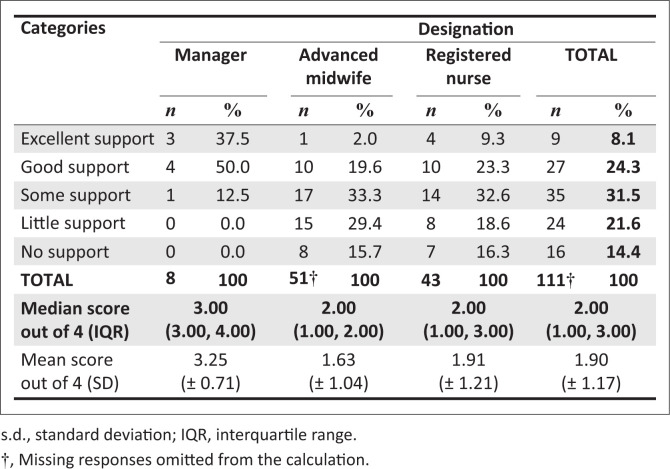
Perceptions of support by designation.

With regard to designation, there was a significant difference in perceptions of support between groups (*p* = 0.0037). Further exploration revealed that managers perceived the support from district management to be significantly higher (3.00 [3.00, 4.00]) than advanced midwives (2.00 [1.00, 2.00]) (*p* = 0.0018) and registered nurses (2.00 [1.00, 3.00]) (*p* = 0.0115) did. Although registered nurses felt they were receiving more support from district management than advanced midwives did, this was not statistically significant (*p* = 0.3684). Figure 5 shows the differences in perceptions of support received by the three main designations.

Neither the age of participants nor the length of their employment at a CLEVER facility significantly influenced perceptions of support received from district management (*p* = 0.1485; *p* = 0.4361, respectively).

### Pandemic preparedness: Qualitative findings

Three main themes emerged from the analysis of the healthcare providers’ questionnaire responses and the field notes and reflective notes of the CLEVER team members. The first theme relates to the working environment and the health system’s readiness for the pandemic. The second theme focuses on quality of patient care and service provision, with special reference to the perceived impact of the pandemic on HCWs’ ability to continue with the positive practice changes made as a result of the CLEVER Maternity Care programme. The third theme is built around HCWs’ response to the pandemic. The second theme gives insight into the reasons for participants’ scoring on the question related to the maintenance of the practices introduced through the CLEVER programme. The first and third themes highlight two of the important support needs identified by HCWs, namely support for a better working environment and personal support, respectively.

Table 1 provides an overview of the themes, subthemes and categories in the framework with a small number of direct quotations from HCWs’ responses. Online Appendix 1 contains a more detailed selection of referenced verbatim quotations to further support the credibility of the findings. Direct quotations from the observations and feedback received by the CLEVER team are included in the discussion of the three themes to follow. In the references for direct quotations ‘M’ refers to an MOU and is followed by ‘A’ to ‘J’ for each of the 10 MOUs followed by the number of the study participant. The style for DH is ‘H’ followed by ‘A’ to ‘D’ plus a number. In the case of the CLEVER team’s field notes, there is no number in the reference.

**TABLE 1:** Framework with themes, subthemes and categories.

Theme	Subtheme	Category	Quotations
**Working environment and health-system readiness**	Planning and management	General	‘If such [*a pandemic*] happens, there must be a proper plan and the plan must be consistent.’ (HC14)
	Resource availability	General resources (material resources; infection prevention and control; equipment) PPE and protective clothing (availability; quality; use; masks) Human resources (staff shortages; staff under quarantine; increased workload; recommendations)	‘If such [*a pandemic*] happens, there must be a proper plan and the plan must be consistent.’ (HC14) ‘Need more PPE and to be able to change the mask after you have taken it out, like after eating not to wear the same mask. When leaving to go home to wear a clean mask and dispose the one that you have been wearing.’ (HB16) ‘Disruptions when staff tested positive and contacts had to be isolated made it difficult to maintain because you had to use staff from other departments to fill in.’ (ME08)
	Infrastructure	Structural constraints (overcrowding; lack of space; screening and isolation facilities)	‘In my facility there is no space, any 4 beds for both patients in labour and post delivery. Social distancing is impossible. People cannot be nursed on the floor, so they are forced to share single bed with two to three patients.’ (MG05)
	Protocol development and patient management	Changes and adherence to routine and new protocols (screening and testing; management PUIs and COVID-19 positive patients; infection prevention and control; mask wearing; social distancing)	‘Changed procedure on how to treat the patient with regard [*to*] giving informed decision regarding covid.’ (MB02)
**Quality of patient care and service provision**	General care	Quality of general and unspecified care (maintained; deteriorated) Increase in waiting times	‘Patient care in maternity was maintained. Casualty work and patient care deteriorated during the pandemic as we couldn’t cope. No new changes implemented.’ (HA02) ‘Waiting times for patient are longer because they have to queue outside the clinic, get screened before they can get their files and got to consulting rooms.’ (MD01)
	Maintenance of CLEVER components	General Support visits not maintained Emergency obstetric drills not maintained Patient support (birth companions; family support Decreased communication with patients and social distancing Labour care (maintained; deteriorated) Respectful care (maintained; deteriorated) Ward rounds and patient handover (maintained; changed) Collegial support and teamwork (positive; negative)	‘At times we are unable to maintain clever maternity care project because if the ward is full difficult to maintain.’ (MA08) ‘No more visits from the district, no more drills and this compromised our services as we still expected to learn from and with them.’ (HB08) ‘No … more drills and this compromised our services as we still expected to learn from and with them.’ (HB08) ‘No more doulas during labour. Some patients need moral support and visitors during their stays.’ (MB03) ‘Communication with patients minimised. Had to maintain a mandatory social distance, had no proper masks (N95).’ (HB01) ‘Pain medication was … given as necessary. … Patients were still allowed to mobilise during labour. They were also allowed to eat and drink as they wished.’ (ME06) ‘As a result of anxiety/fear of staff I have noticed that some staff members treat patients in a more mean way than usual. (HD05) ‘Patient handing over maintained.’ (HB02) ‘However deteriorating in terms of respect amongst each other as some were not coping because of their various personal issues.’ (HD06)
**Healthcare workers’ response to the pandemic**	Fear	Fear of transmission (fear for colleagues; fear for and of patients) Reactions to fear (panic; denial; towards colleagues; towards patients; calmness	‘There was so much to be fearful of, less contact with patient relatives and patient included.’ (HB03) ‘[*A*]nd the increase of death that made all of us to start panicking.’ (HC06)
	Lack of information and communication	Effects of lack of information Recommendations on education and training (general; dealing with the pandemic and patient management; PPE training; Essential Steps in Managing Obstetric Emergencies (ESMOE); patient education)	‘There’s been a lot of change of information about the virus …’ (HC06) ‘More information on how to deal with the pandemic.’ (HC06)
	Perceptions of support received	Perceived support from management and district coordinators (positive and negative) Psychological / emotional support and appreciation	‘Minimal support was maintained by Tshwane district coordinators.’ (MF06) ‘We … managed to talk about our feelings with psychologist.’ (HA05)
	Demands for mental health and financial support	Need for acknowledgement and appreciation; debriefing Compensation	‘I think emotional support is always key for health workers. They are also social beings who over and above also experience challenges in their personal life – in addition to the work related challenges.’ (HB14) ‘We deserve compensation [monetary form] because we never got a salary increase this year. A COVID-19 risk related compensation will really be welcomed.’ (HA02)

PPE, personal protective equipment; COVID-19, coronavirus disease 2019; PUI, person under investigation; ESMOE, Essential Steps in Managing Obstetric Emergencies.

Note: Participant identifiers: M, MOU, A to J, 10 MOUs followed by the number of the study participant; H, district hospitals, A to D, 4 district hospitals followed by the number of the study participant.

#### Theme 1: Working environment and health-system readiness

We identified four subthemes around the ability of the healthcare system to respond to the pandemic in a timely manner at various levels of the system: general planning and management; resource availability; infrastructure; and protocol development and patient management.

With regard to *planning and management*, CLEVER team members reported on the major role of management and leadership in getting a facility ready for the pandemic. Facilities that had difficulty with pandemic readiness had ‘absent’ (MI), ‘not available’ (MD) or ‘hands-off managers’ (HC) and there was ‘no cohesion between managers on different levels’ (HC), ‘no support from management’ (MI) and ‘not a lot of support from management above’ (HB). One facility had a ‘hands-on’ manager, but there was also ‘poor communication flow between manager and labour ward’ (ME). Three facilities had ‘the support of a family physician’ (MF, MH and MI).

*Resource availability* is interlinked with planning and management. Survey participants generally mentioned deficits or shortages with regard to three types of resources that influenced the way they could function: general resources such as equipment and infection prevention and control (IPC) materials (soap, sanitiser, paper towels); PPE and clothing; and human resources.

Availability of sufficient PPE was one of the most dominant recommendations from survey participants. On the other hand, the CLEVER team observed that PPE was not always used even when available; the reason given was the ‘hot weather’ (MI; MJ). Masks seem to have been less of an issue, although individuals from some facilities felt they were insufficient. Participants from one MOU and one district hospital reported having sufficient PPE.

The already critical human resource shortage in most facilities was highlighted after an ‘overwhelming influx of patients’ (HD17) whilst some facilities were closed because of COVID-19 outbreaks with staff members in quarantine or in isolation. One district hospital was particularly hard hit because it had to absorb deliveries from another district hospital that had been converted into a COVID-19 designated hospital.

Participants from some of the study facilities referred to poor *infrastructure* and structural constraints that led to overcrowding and hampered their service and care efforts. These were exacerbated by more general problems observed by the CLEVER team such as ‘no water for the past two days’ (HC), ‘no linen’ (MA), malfunctioning labour equipment and generator (MA; MG; MH), broken delivery beds and stock-outs of CTG paper, HemoCue slides, PCR kits, pethidine, linen savers and *Road to Health* booklets.

One district hospital found creating appropriate isolation facilities a challenge. The observations by CLEVER team members confirmed the lack of space to create isolation rooms in maternity units and to maintain social distancing in a number of the MOUs and DHs (HA; HB; MJ): ‘No isolation room for MOU patients and staff did not know where to place a suspect who is in labour because of infrastructure’ (MD). Where space was available, there was sometimes a lack of preparedness: ‘One delivery room has been identified as the isolation room, but … not prepared. … Staff in MOU trained and aware but were not ready to receive a suspect’ (MH).

Survey participants commented on adherence to routine and *new protocols* and their ability to adhere to these protocols in the *management of patients*. Healthcare workers in some facilities reported that they were able to maintain previous protocols and adhere to new ones, but those in other facilities found adherence more difficult.

There were many comments on screening and testing. By the time the questionnaire was administered, most facilities had streamlined their screening and other precautionary measures. The CLEVER team members observed that ‘patient screening was not conducted in the beginning’ (HA); ‘the screening of patients in labour was not appropriately performed’ (HB); and ‘no spraying at the gate, no screening’ (MD). By September 2020 at least one MOU was still failing to screen patients, whilst the staff did not wear PPE, only masks. There were also differences between facilities regarding where and by whom the screening was performed. In some facilities screening was carried out only at the general entrance (MD; ME; HB), and in others it was performed at the labour ward entrance (MB; HC; HD). In some facilities security staff or quality assurance personnel did the screening; in others ‘the screening is performed by the midwives’ (MH; MB; MI; HC; HD).

With regard to testing, a number of HCWs were dissatisfied with the absence of regular testing of staff members and felt unsupported by the health authorities. The majority of participants claimed that general IPC measures were being maintained. There were a few negative comments from MOU participants on cleanliness of wards and issues with deep cleaning when required.

#### Theme 2: Quality of patient care and service provision

There are two subthemes related to patient care and service provision. The first theme focuses on general care, and the second theme relates to the maintenance of the components of the CLEVER Maternity Care programme.

Healthcare workers had varying views on whether and to what extent the *general quality of care* could be maintained in the light of the additional demands and stresses placed on the system by the pandemic. The majority of participants believed that they could maintain their standards of care.

With regard to the *maintenance of the positive changes* brought about by the *CLEVER Maternity Care* programme, participants held different views, some more positive than others. The CLEVER components mentioned by participants included the following: support visits combined with emergency obstetric drills; support for patients; communication with patients; labour care; respectful care; ward rounds and patient handover; collegial support and teamwork. As a result of the lockdown and COVID-19 regulations, support visits from the CLEVER team, which was also responsible for conducting emergency obstetrics drills, were curtailed.

The new regulations did not allow women in labour to have a labour companion or to receive visitors for support. Social distancing and inadequate PPE also led to diminished communication with patients and made patients feel neglected. Participants generally indicated that respectful care had been maintained. Ward rounds and patient handovers as modelled in the CLEVER Maternity Care outreach seem to have been maintained in some facilities but there were changes in others. The open-ended responses give an indication why the majority of survey participants felt that all, most or at least some practices (76.8%) introduced through the CLEVER programme could be maintained.

The CLEVER team observed a ‘united staff team’ (MF), ‘midwives working as a team’ (MB) and ‘strong teamwork and support’ (HB) during their visits to some of the facilities. At one MOU ‘teamwork is there and they support each other, however, no leadership in the MOU’ (MI) and at one district hospital ‘midwives support each other even though conditions are not appealing’ (HB). At another hospital ‘strong teamwork’ (HC) was observed amongst midwives, but there was ‘no cohesion between managers on different levels’ (HC). In other facilities teamwork was observed to be ‘not optimal’ (MA; MC), with a ‘discordant team of midwives … where night staff [are] not communicating with day staff’ (MC) in one MOU. Some HCWs reported positively on the teamwork in their facility but in others a decline in morale and deteriorating respect for each other were observed.

Based on the observations during support visits, we developed the following typology of teamwork during the pandemic:

- Teamwork amongst maternity staff with facility manager on board (ME; MH; HD)
- Teamwork amongst maternity staff with facility manager not part of the team (MB; MJ)
- No teamwork in maternity with a facility manager who is trying her best (MC)
- No teamwork in maternity and no support from facility manager (MI)

#### Theme 3: Healthcare workers’ response to the pandemic

The first theme provided background information on the preparedness, functionality and ability of HCWs to deal with the pandemic. Four subthemes were identified that reflected their response to the pandemic: fear; lack of information and communication support; perceptions of support received; and demands for debriefing and compensation.

The advent of COVID-19 meant that little information was available and communication with survey participants was infrequent. They *feared* for their own health and that of their colleagues and were afraid that patients might be infectious. Participants made a number of recommendations related to improved *communication and information*. These included the following: general requests; dealing with the current epidemic and future pandemics; more information on COVID-19; management of COVID-19 positive patients; and patient mobilisation and education.

The stressful working environment has already been described in more detail under theme 1, especially in terms of resource availability and adherence to new protocols that required additional work. The CLEVER team regularly observed staff exhaustion (MH, HD). Healthcare workers expressed a strong desire for *support and appreciation* from managers and health authorities, especially in the district hospital that had to take over the patients from the neighbouring hospital without additional staff. ‘Some staff want to leave because they say they don’t get support from management’ (HD). Participants at one district hospital did, however, acknowledge the comprehensive support they received. Healthcare workers also missed the regular support visits ‘for helping us, not inspecting’ (MA10).

Flowing from the perceived lack of support, two needs were highlighted – a need for *mental health support* and a need for *compensation*. Psychological support needs were expressed through terms such as ‘debriefing’, ‘counselling’, ‘emotional support’, ‘mental support’ and ‘wellness clinic’. For some participants, individual counselling instead of group counselling only was important. Participants’ demands for ‘remunerative appreciation’ (MA11) stem from a desire for recognition from the health authorities for their dedication and hard work. Most of the rewards suggested were some form of financial compensation such as a danger allowance, a salary increase or extra leave.

### How ready were the health facilities for the pandemic?

**FIGURE 6:**
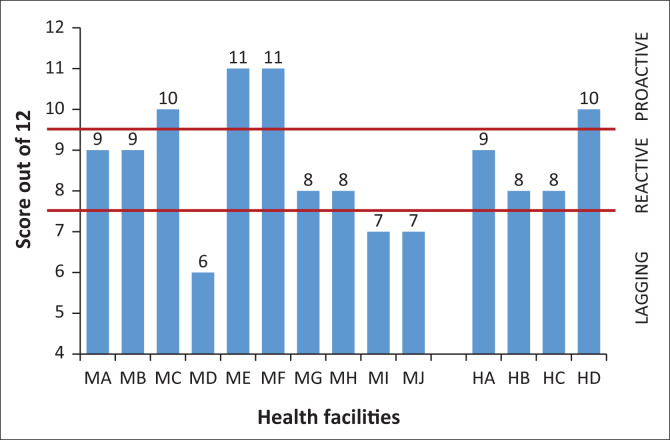
Subjective ranking of health-facility pandemic readiness.

The researchers identified three categories to create a typology of health-facility pandemic readiness: proactive, reactive and lagging facilities. In proactive facilities HCWs immediately started preparing to protect their staff and to receive confirmed COVID-19 patients or PUIs safely. Reactive facilities tended to do nothing until staff were prompted or ran into problems. Lagging facilities tended to be somewhat ‘paralysed’ despite receiving directives to make changes. The rankings of the four researchers with regard to pandemic readiness were added together to get a score out of 12. For each proactive ranking a facility scored 3, for each reactive ranking 2 and for a lagging ranking 1. Figure 6 gives a visual presentation of the rankings and the facility scores. A score of 10–12 indicates a proactive facility, a score of 8–9 a reactive facility and a score of 4–7 a lagging facility. Three MOUs and one DH were evaluated as proactive, four MOUs and three DHs as reactive and the remaining three MOUs as lagging.

## Discussion

This study aimed to describe and understand how the pandemic influenced the gains made by an initiative to improve the quality of obstetric service provision and respectful care. We illustrated how maternity HCWs perceived and experienced changes in protocols and practice as a result of the pandemic. Service provision pressures were similar to those in other countries.^13,14^ The needs and challenges that HCWs expressed in this study indirectly advocated for a resilient health system in which managers provide clear communication, attend to adequate risk management and resources and are supportive of their mental health needs and well-being.^17,20,21^

In this study maternity HCWs across the board had similar perceptions and experiences of changes in practice and services and the support they received from district management. Managers, however, were more positive about the support they had received than advanced midwives and registered nurses. Because of their involvement in procurement and pandemic response, managers received more direct communications from district management that did not trickle down to the frontline HCWs. Additional pressure on advanced midwives as senior professionals leading the labour ward could explain their low score regarding support received. There was less leadership capacity for essential services as some of mid-level managers were moved to COVID-19 duties.^11,14,22^

Although most survey participants considered it impossible to maintain all the positive changes enabled by the CLEVER Maternity Care outreach, more than three quarters felt that at least some of the good practices were maintained. Midwife-led obstetric unit participants scored the maintenance of CLEVER practices significantly higher than those from DHs. This could be explained by the higher COVID-19 patient load and increase in deliveries in the DHs, which was also experienced in other parts of South Africa.^14^ Important elements of the CLEVER programme include knowledge sharing, teamwork and the building of skills and capabilities.^12^ Although the full programme could not be completed, the CLEVER support team was able to maintain some positives during the surge of the pandemic through support visits that included information sharing and communication, problem-solving, discussing changes to regain optimism, encouraging HCWs to continue with ward rounds and teamwork and giving feedback to managers. Activities of this nature were also reported in the literature as a means of building a more resilient, capable health workforce^21,23,24^ and could be used by other healthcare providers and leaders to boost morale and maintain quality care.

The three themes in the interpretation framework of this study could be interpreted according to the health-systems building blocks.^14,17^ The first theme of working environment and health-systems readiness relate to the health workforce’s ability to provide services and to experiences of how the functioning of the other components of the health system affected the safety and quality of patient care. Participants’ comments on resource availability and infrastructure pertain to elements of service delivery, access to essential supplies and health financing. The second theme of quality of patient care and service provision falls within the ambit of service delivery and clinical governance. Healthcare workers’ response to the pandemic (theme 3) demonstrates the perceived neglect of the needs of the health workforce, especially regarding support for mental well-being. Participants’ demands for compensation have implications for financing, whereas lack of information and communication has some bearing on health information systems.

Facilities needed to respond swiftly to the pandemic. As one size does not fit all, fulfilling support needs had to match the gaps in the local health system. For this reason we developed a typology for describing the pandemic readiness of health facilities (proactive, reactive and lagging), which was underpinned by the research team’s reflections according to the health-systems building blocks. It could be a useful categorisation for describing health facility readiness during future pandemics and for managers to tailor their support to individual facilities. Similar typologies were also developed elsewhere in the world during the pandemic. For example, Cay and Panganiban developed a typology based on the health-systems building blocks to categorise the status of the health system and resilience of public hospitals in Batangas, Philippines. Their typology also comprises three categories: basic, developing and progressive.^21^

The CLEVER Maternity Care programme appears to have mitigated some of the anticipated areas of deterioration in service delivery and the majority of survey participants indicated that it has been possible to maintain many CLEVER practices despite the pandemic. On the other hand, one possible reason for their perceptions of lack of support and information could be that senior managers were overloaded with screening, contact tracing and pandemic monitoring. They were caught off guard, could not keep up with their day-to-day responsibilities and were therefore unable to provide sufficient support with the management of resources, the monitoring of patient care and service provision, and communication and debriefing. Worldwide, unprecedented actions were needed to streamline resources to sustain essential health services pertaining to infrastructural and infection control difficulties in LMICs.^7,25^

### What did we learn from our experience during the pandemic?

One of the main lessons from this pandemic was the crucial role expected of mid-level managers such as family physicians, primary healthcare and sub-district managers, coordinators and programme managers in the public health sector. They did not realise that they were next in line to lead with regard to patient safety, risk management, communication and management of resources, whilst senior managers were being deployed to manage the pandemic. Mid-level managers have a unique knowledge of the local pathways and should have been able to manage adjustments to provide services under stressful conditions.^24,26,27^

Patient safety and risk management entail that the workforce has to share responsibilities in reducing the impact of the pandemic, develop core competencies and follow standardised protocols and guidelines to prevent a failure in service delivery.^28^ A lesson from our study was the uncertainty created by the non-communication of new COVID-19 protocols and guidelines (see participant quotes in Online Appendix 1). Adherence to new protocols and routines should be discussed and explained at all sub-levels of the health system to raise awareness and aid implementation. Managers also have a role in reviewing gaps in service provision emanating from the pandemic. For example, HCWs viewed patients as a vector of infection, leading to distant care provision with less interaction that impacted the quality of care.

In addition to patient safety, managers are also obliged to look after the safety of staff. This was a very prominent response in our study, especially with regard to the provision of adequate PPE. Staff need to feel safe and a breakdown in trust leads to a low morale.^29^ Mid-level and subdistrict managers, family physicians, coordinators and programme managers are best placed to promote resilience in the health workforce, thereby aiding the pandemic response.^27^ According to our study and the international literature there was an increase in HCWs with compassion fatigue and psychological distress, depression and anxiety and this limited their capabilities of caregiving.^15,23,30^ Facility managers have a role to reassure staff and promote resilience. They can, for example, activate the occupational health and safety teams to review HCWs’ well-being and work morale and to provide feedback and support with appreciation during recovery sessions.^24,29,30^ Group sessions taking place on-site would greatly alleviate mental health distress^29^ and could bring back trust in the health system.^21,30^

### Study’s strengths and limitations

This study provided a small snapshot at the primary care level in one health district in South Africa of how the COVID-19 pandemic had influenced experiences of service provision during the first six months of the COVID-19 pandemic. Although a convenience sample of just over 100 participants was obtained in the HCW survey, the participants’ self-reports and the themes that emerged align with the findings of other studies across the world.^4,6,9,14^ Some of the findings of this study could therefore be transferable to similar settings in South Africa.

Various other measures illustrate the trustworthiness of the study.^31^ Actions contributing to the credibility of the study included a period of 5 months of site visits (July 2020 to December 2020), the constant engagement of four researchers in the data analysis and interpretation of findings and the verbatim quotes from the CLEVER team’s field notes and from study participants, reflecting both positive and negative responses (Table 1 and Online Appendix 1). The use of multi-methods to triangulate perspectives from maternity HCWs and the CLEVER implementation team and the 96 site visits by the team enhanced the dependability of the study. The diverse professions in the CLEVER team and the weekly reflective discussions of the visits assisted as cross-checks for researcher subjectivity, thus contributing to confirmability.

The ranking of facility readiness was based not on an objective checklist but on the subjective impressions of the researchers. The ranking was part of an in-depth discussion on the support needs of facilities that cannot always be captured with a checklist. It also guided decision making in terms of what could be performed locally and what was beyond the abilities and power of people at the grassroots level.

## Conclusion

The study illustrated how a pandemic can bring programmes in the process of implementation to a slow-down or halt, necessitating the rethinking of implementation methods. The CLEVER programme had to deviate from its original implementation plan by using different opportunities to model care with innovation in order to achieve the initial goals. This entailed helping HCWs to accept the ongoing pandemic, find a way to quality care and learn from failures. Support to the healthcare workforce to provide quality health services should include advocacy for knowledge sharing, teamwork, networking and building of skills and capabilities to reach pandemic readiness.

Feedback to the district management team is important in a pandemic. Our study illustrated how one could elicit meaningful information to assist the team in planning strategies to mitigate the impact of the pandemic on service provision. The lessons learned from this pandemic could be used to build responsive and resilient primary healthcare systems that are supported by leadership that understands collective problem-solving to reconfigure scarce resources.
